# CancerDiscover: an integrative pipeline for cancer biomarker and cancer class prediction from high-throughput sequencing data

**DOI:** 10.18632/oncotarget.23511

**Published:** 2017-12-20

**Authors:** Akram Mohammed, Greyson Biegert, Jiri Adamec, Tomáš Helikar

**Affiliations:** ^1^ Department of Biochemistry, University of Nebraska-Lincoln, Lincoln, Nebraska, United States of America

**Keywords:** open-source, cancer classification, gene expression, machine learning, cancer biomarker

## Abstract

Accurate identification of cancer biomarkers and classification of cancer type and subtype from High Throughput Sequencing (HTS) data is a challenging problem because it requires manual processing of raw HTS data from various sequencing platforms, quality control, and normalization, which are both tedious and time-consuming. Machine learning techniques for cancer class prediction and biomarker discovery can hasten cancer detection and significantly improve prognosis. To date, great research efforts have been taken for cancer biomarker identification and cancer class prediction. However, currently available tools and pipelines lack flexibility in data preprocessing, running multiple feature selection methods and learning algorithms, therefore, developing a freely available and easy-to-use program is strongly demanded by researchers. Here, we propose CancerDiscover, an integrative open-source software pipeline that allows users to automatically and efficiently process large high-throughput raw datasets, normalize, and selects best performing features from multiple feature selection algorithms. Additionally, the integrative pipeline lets users apply different feature thresholds to identify cancer biomarkers and build various training models to distinguish different types and subtypes of cancer. The open-source software is available at https://github.com/HelikarLab/CancerDiscover and is free for use under the GPL3 license.

## INTRODUCTION

Classification of a tissue sample as cancer or normal and among different tissue subtypes facilitates cancer treatment, and high-throughput techniques generate massive amounts of cancer data. Machine learning (ML) has the potential to improve such classification, and the traditional motivation behind ML feature selection algorithms is to find the optimal subset of features. However, no single feature selection algorithm or classification algorithm can provide the best set of features and classifiers [1]. Therefore, there is a need to develop a pipeline that lets users apply different feature selection algorithms, feature thresholds, and various classification algorithms to generate accurate prediction models and evaluation reports that distinguish cancer from normal samples, as well as different types and subtypes of cancer.

Remarkable efforts have been put to develop gene expression analysis tools and databases for cancer high-throughput data [2–11]. Several machine learning tools have been developed to study cancer classification [12–16]. However, Classifusion [14], ESVM [15], Prophet [16] are either not available, abandoned or not maintained. The available platforms require processed raw data that have been normalized to address various technical and statistical challenges such as, gene expression value differences within the datasets and sequencing platform bias. Moreover, different analysis steps have to be performed manually by various tools, often using different software platforms. These long and manual processing steps are not only time-consuming but also error-prone, making high-quality, large-scale ML analyses difficult.

To this end, we have developed CancerDiscover, an integrative software pipeline, which, given raw, bulk high-throughput data from various platforms, can perform quality checks, normalize the data, select the most important features from multiple feature selection algorithms, and build and evaluate different machine learning models in a streamlined fashion. Unlike software tools that require manual processing and are limited in feature selection and classification algorithm options (e.g., GenePattern [13] and Chipster [12]), CancerDiscover is a fully automated pipeline, while providing users with full control over each analysis step. CancerDiscover is complementary to the data repositories, data visualization, and the software tools such as ONCOMINE [9], INDEED [10] and cBioPortal [11] that support data visualization and analysis of differential gene expression. Herein, we describe the open-source software and demonstrate its utility and flexibility through a case study. We also demonstrated the utility of CancerDiscover pipeline using 2,175 gene expression samples from nine tissue types to identify tissue-specific biomarkers (gene sets) whose expression is characteristic of each cancer class and built single-tissue and multi-tissue models [17]. In the end, we provided the benchmarking statistics using CancerDiscover for datasets of varying sizes.

## RESULTS AND DISCUSSION

### Implementation

The purpose of CancerDiscover pipeline tool is to allow users to efficiently and automatically process large high-throughput datasets by transforming various raw datasets and selecting best performing features from multiple feature selection algorithms. The pipeline lets users apply different feature thresholds and various learning algorithms to generate multiple prediction models that distinguish different types and subtypes of cancer.

**Figure 1:**
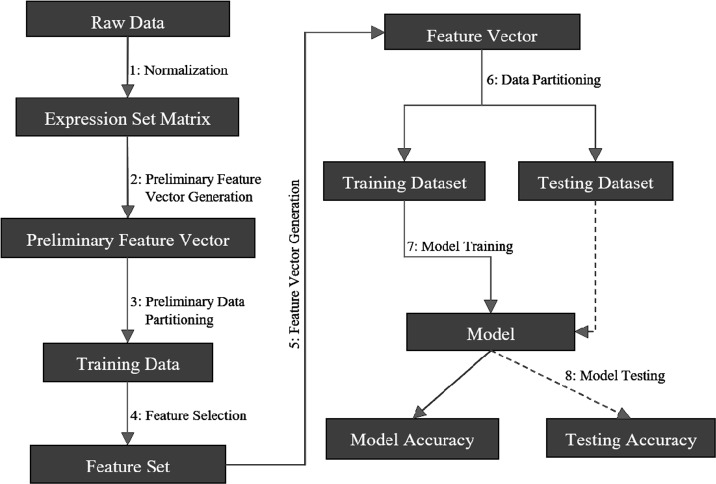
Schematic representation of the CancerDiscover pipeline First, raw data are normalized, background correction is performed, and the output is partitioned into training and testing sets. The test set is held in reserve for model testing while the training set undergoes a feature selection method. Feature selection provides a list of ranked attributes that are subsequently used to rebuild the training and testing sets. The training dataset is subsequently used to build machine learning models. Finally, the testing data set is used for model testing.

CancerDiscover takes raw datasets, normalizes it, generates WEKA [18]-native (Attribute-Relation File Format: ARFF) input files. The pipeline is illustrated in Figure 1. CancerDiscover consists of eight components: normalization, preliminary feature vector generation, preliminary data partitioning, feature selection, feature vector generation, data partitioning, model training and model testing. These components are organized into three scripts (masterScript_1, masterScript_2, and masterScript_3). In addition to Bash, the CancerDiscover pipeline is also implemented in SLURM (Simple Linux Utility for Resource Management) to make it available for high-performance computing clusters.

(1). **Normalization:** Due to the inherent differences among samples obtained from various studies, normalization and background corrections are required to remove or subdue bias in raw data for accurate models. Once raw high-throughput data is obtained, normalization and background corrections are performed to remove the technical variation from noisy data and background noise from signal intensities and generated the expression set matrix (for example, gene expression intensity values, etc.).

(2). **Preliminary Feature Vector Generation:** Next, the expression set matrix is used to create the master feature vector in the WEKA-native file format (ARFF).

(3). **Preliminary Data Partitioning (Stratified):** Stratified data partitioning was used by splitting the master feature vector into training and testing sets to maintain an even distribution of class distribution. These training sets are used to construct the models after feature selection has been performed in the next step. Later, the model's accuracy will be assessed with the testing set, which had not been exposed to the model, giving an honest assessment of the model. Users of CancerDiscover can specify the size of the data partition of their choice in the pipeline.

(4). **Feature Selection (on training data set only):** Our pipeline offers the ability to select multiple feature selection algorithms. Each of these algorithms provides the list of ranked features that distinguish different types and subtypes of cancer. Users can choose different feature thresholds and can explore the relationship between the number of features considered by the classification algorithms and model accuracy. For example, the feature sets generated can be separated into different feature thresholds (including the top 1%, 10%, 33%, 66%, 100% of the total number of ranked features as well as the top 25, 50 and 100 ranked features). Users can also arbitrarily choose these thresholds to identify the minimum number of features needed to achieve accurate classification models. For a list of available feature selection methods, see Supplementary File 2.

(5). **Feature Vector Generation:** Since the classification models must be built based only on the ranked features, new feature vectors are generated based on the ranked feature sets.

(6). **Data Partitioning (Stratified):** Once the new feature vectors are generated, each feature vectors file will undergo a second data partitioning. This partitioning seed value (or integer that defines the exact sequence of a pseudo-random number) is the same as the one used in the preliminary data partitioning. As such, each new feature vector will be split into the same training and testing sets as in step 3. The master training and testing feature vectors and the new training and testing feature vectors differ only in the number of features; the master feature vectors contain all of the features, whereas the newly created feature vectors contain only the features that ranked according to different thresholds (Dimensionality Reduction).

(7). **Model Training:** CancerDiscover provides a diverse set of machine learning classification algorithms and allows the user to build models as they see fit. Each new training dataset from the above step undergoes machine learning model construction using stratified k-fold cross-validation to identify the optimal model.

(8). **Model Testing:** The model performance was assessed by testing its accuracy using the testing dataset that was kept hidden from the model. The model can also be used to predict the class labels for the samples in the unknown dataset. In the case study below, we illustrate the utility of the software to classify normal vs. cancerous tissues and adenocarcinoma vs. squamous carcinoma based on gene expression data.

### Installation/operation

CancerDiscover software is available at https://github.com/HelikarLab/CancerDiscover All the components of the pipeline are organized into three scripts (namely masterScript_1, masterScript_2, and masterScript_3), each of which is composed of several scripts (PERL, AWK, SHELL, BASH, R, and SLURM). The detailed installation/operation of the pipeline is described in the Supplementary File 1. There are two versions of the CancerDiscover pipeline: the beginner version consists of bash scripts that can be run on the local machine and an advanced version that consists of SLURM (Simple Linux Utility for Resource Management) scripts that can be run on a high-performance computer (HPC). SLURM is a computational architecture used to organize user requests into a queue to utilize high-performance computing resources. Due to the sheer size of the high-throughput data and complexity of data processing steps, it is recommended to use CancerDiscover on a high-performance compute cluster. The command-line pipeline is compatible with Linux OS and Mac OSX.

### Case study

**Figure 2:**
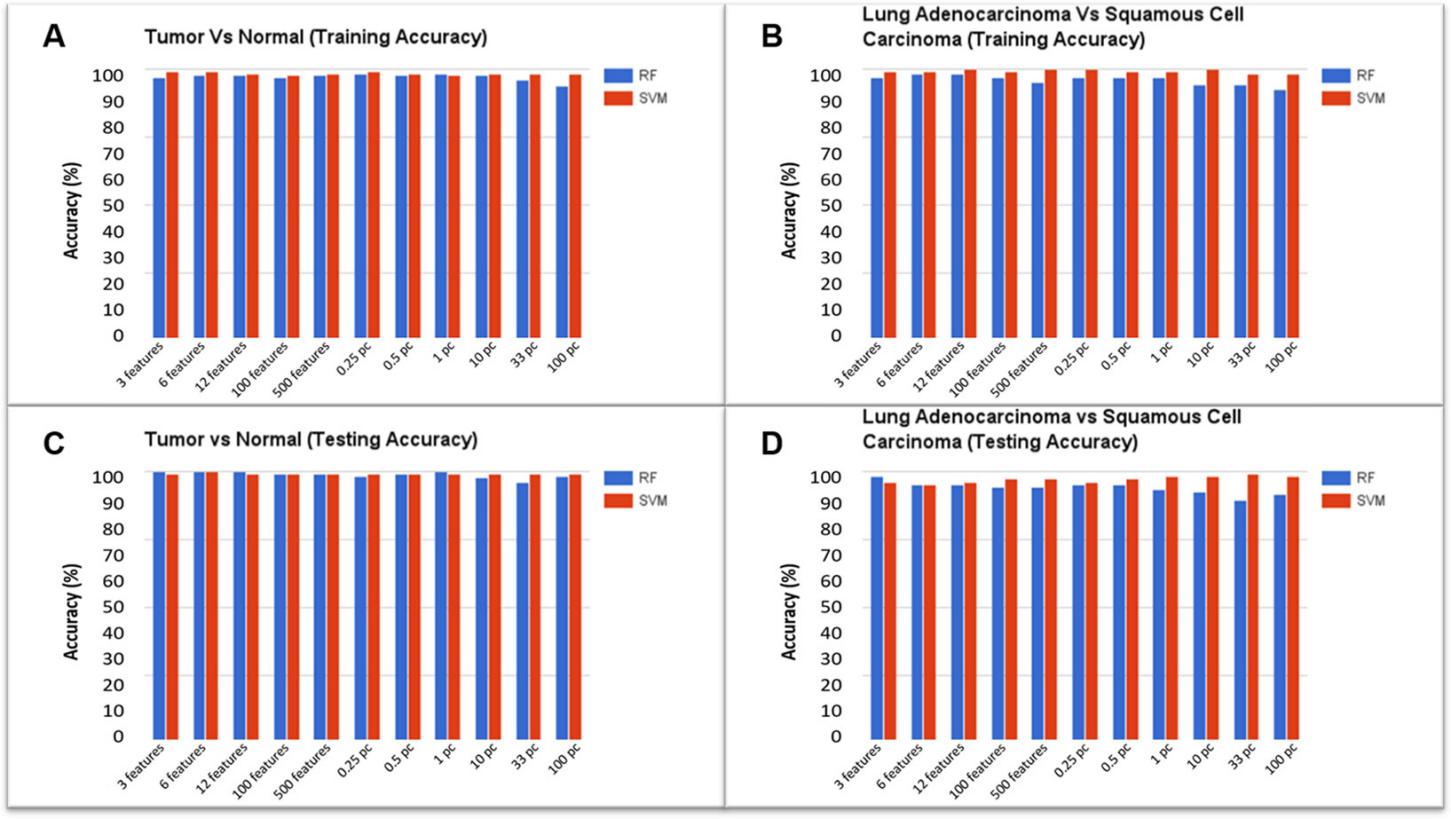
Model accuracies for the classification of tumor vs. normal and adenocarcinoma vs. squamous cell carcinoma: RF represents Random Forest classifier and SVM indicates Support Vector Machine classifier **(A)** Training accuracy for Tumor vs. Normal model, **(B)** Training accuracy for Adenocarcinoma vs. Squamous Cell Carcinoma model, **(C)** Testing accuracy for Tumor vs. Normal model, **(D)** Testing accuracy for Adenocarcinoma vs. Squamous Cell Carcinoma model.

Two kinds of ML models were generated and tested to illustrate the possible application of the pipeline. The first model was developed to classify tissue samples as either cancerous or normal, according to their gene expression patterns. Sample distributions were as follows: 237 tumor tissue samples and 17 histologically normal tissue samples split evenly into testing and training data sets. The Quantile Normalization Method [19] was used to normalize the data, and the background correction was performed using the Robust Multichip Average (RMA) [20] parameter method by modifying the configuration file for the case study presented in the paper. Filtered Attribute Evaluator combined with Ranker method was the algorithm selected (using pipeline configuration file) to perform feature selection on the training dataset. This algorithm outputs a list of all data features ranked according to their utility in distinguishing the different classes of samples; features ranked at the top of the list are most useful in distinguishing cancer from normal samples. The plates used for this case study contain approximately 10,000 full-length genes corresponding to 12,625 probes (features). The top (0.25%, 0.5%, 1%, 10%, 33%, 100%) ranked features, as well as additional feature sets containing the top (3, 6, 12, 100, 500) features were used for generating several models simultaneously. Training and testing accuracies are reported in Figure 2A-2D. We selected RF and SVM as the machine learning classification algorithms for the case study.

We achieved a model training accuracy of 98.43% for the RF classifier using the top 0.25% (31 attributes) of features. Models constructed using the top 3% of ranked features reported an accuracy of 96.06%, while models using the entire list of features (100%) resulted in the lowest accuracy of 93.70%. Training accuracies for the SVM classifier were 99.21% for the models that used the top 3 features. Accuracy declined with the increasing number of features, with models that used the top 12 ranked features reporting an accuracy of 98.43%. SVM resulted in the lowest (though still relatively high) accuracy of 97.64% using 100 features. As few as the top 31 features are sufficient to achieve a higher accuracy, using random forest classifiers, whereas top 3 features are sufficient to achieve a higher accuracy using support vector machines.

The second set of models was also bi-class; however, the models were developed to distinguish lung sub-types (adenocarcinoma vs. squamous cell carcinoma), rather than tumor vs. normal tissue. 211 lung adenocarcinoma and squamous cell carcinoma samples were evenly split into training and testing datasets. After feature selection, the list of ranked features was used to generate models based on different feature thresholds. Results from testing accuracies can be seen in Figure 2B. With the entire list of ranked features, the RF testing accuracy was 91.51%, increasing in accuracy as the percentage or number of ranked features decreased. The top 1% of ranked features (126 attributes) resulted in a model testing accuracy of 93.40% while the top 0.25% (31 attributes) of ranked features resulted in testing accuracies of 95.28%. A similar trend was seen going from the top 500 features to the top 3 features. On average, SVM testing accuracies were more consistent and higher than those based on RF. The model generated with top 3 features resulted in an accuracy of 96.23% while using the top 6 features led to the accuracy of 95.28%. Using 100 features resulted in a testing accuracy of 97.17%. Using the top 0.25% and 0.5% resulted in accuracies of 96.23% and 97.17%, respectively, while using the top 1% and 10% features led to an accuracy of 98.11%. Using the top 33% of ranked features resulted in the highest testing accuracy of 99.06%. Precision, Recall, and F1-Score for the models generated using the top 3 features are reported in Table 1.

**Table 1:** Accuracies of random forest models using top 3 features

Training model	Precision	Recall	F1-Score
**Tumor vs. normal**	98.3	98.3	98.3
**Adeno vs. squamous**	97.9	98.9	98.4

As shown in Table 1, we were able to achieve a high degree of accuracy using a small fraction of top-ranked features (3 features). This case study illustrates the pipeline's flexibility, utility, and ease-of-use in the generation of several models simultaneously from raw high-throughput data. It also highlights the customization allowed by CancerDiscover on the individual steps of a typical high-throughput data analysis pipeline, including the data preprocessing, normalization methods, data partitions, feature selection algorithms, classification algorithms, and the threshold or percentage of ranked features for additional analysis.

### CancerDiscover benchmarking

Benchmarking was performed using 500 samples from Acute Myeloid Leukemia (AML) data (See Data Collection section for more details) to assess the performance of the software by running all the feature selection and classification algorithms. The following sample quantities were used; 500, 200, 100, 50 and 10. Each dataset was run through the pipeline performing 23 feature selection algorithms (See Supplementary File 2 for the list of FS methods) and classification algorithms to determine the required computational resources such as the total amount of elapsed time for each step of the pipeline and the total amount of elapsed time. These factors, mainly depend on the size of the dataset being processed. Benchmarking was performed using computational resources at the Holland Computing Centre of the University of Nebraska-Lincoln which has 106 nodes, 4 CPU per nodes. Table 2 below shows the benchmarking results.

**Table 2:** Benchmarking results

Samples	Feature selection methods	Models generated	Normalization (Elapsed Time)	Feature selection (Elapsed Time)	Model train & test (Elapsed Time)	Total
500	20	665	2:05:32	21:45:59	8:05:32	31:57:03
200	20	650	0:52:31	14:16:55	4:49:33	19:58:59
100	20	665	0:26:56	13:31:22	3:12:30	17:00:48
50	20	665	0:16:48	12:06:42	2:58:56	15:12:26
10	19	585	0:07:03	10:05:17	2:14:05	12:26:25

All the datasets contain 54,675 features, and 2 CPUs were used for the analysis.

Elapsed time refers to the amount of real-time spent processing that function.

For the smallest set containing only ten samples, 19 of the 23 possible feature selection algorithms completed processing (4 feature selection algorithms could not be completed due to the 10-fold cross-validation used). For those 19 feature selection algorithms, 585 classification models were generated (few of the ARFF files were empty for the lower feature thresholds due to the small number of samples). The 50-sample dataset completed 20 of the 23 possible feature selection algorithms, thereby generating 665 classification models. When using 100 samples, 20 of the 23 possible feature selection algorithms were completed, and subsequently utilized to generate 665 classification models. The 200-sample dataset provided 20 of the 23 possible feature selection outputs and produced 650 classification models. Lastly, the 500-sample dataset contained 20 out of the possible 23 feature selection outputs and generated 665 classification models. As the datasets grew, the time required for cancer classification increased linearly (Table 2).

### Comparison of CancerDiscover with other methods

We compared the performance of CancerDiscover with that of the three existing methods, GenePattern [13], Chipster [12] and the method described in Aliferis et al. [21]. We used the same train and test datasets to compare the performance of CancerDiscover with these methods. Results of this analysis are summarized in Table 3, Supplementary Table 1 (Supplementary File 3), and discussed in detail below.

**Table 3:** Comparisons of machine learning classification components

Tool Components	CancerDiscover	GenePattern	Chipster	Aliferis
Normalization	✓	-	✓	-
Background correction	✓	-	-	-
Partitioning	✓	-	-	-
Feature selection	✓	-	-	✓
Modeling	✓	✓	✓	✓

This table highlights the capabilities of tools for performing different functions necessary to generate ML models.

GenePattern [13] is a web-based platform that allows users to upload data and perform statistical analysis and class prediction. Due to the nature of the data used in this study, only SVM classification suite was used to draw comparisons between CancerDiscover and GenePattern. Because GenePattern could not perform normalization and background correction for the given datasets, we used the data normalized by the CancerDiscover pipeline (using RMA method) and provided the normalized data to the SVM classification module of GenePattern. The input data contained all probes as GenePattern did not provide feature selection options. ML classification models were generated using the training data with accuracies of 98.43% for the Tumor vs. Normal model, and 99.06% for the Adenocarcinoma vs. Squamous Cell Carcinoma model. These higher accuracies could also be due to the normalization and background correction performed by the CancerDiscover pipeline. Of all the three compared software tools, GenePattern's accuracies are most similar to the ones produced by CancerDiscover – 99.21% and 99.06%, respectively. All probes were utilized in the model building since feature selection could not be performed using GenePattern. On the other hand, CancerDiscover was able to achieve similar accuracy by using as few as three probes (See Supplementary Table 1 in Supplementary File 3). Finally, CancerDiscover differs from the proprietary GenePattern by the fact that CancerDiscover is open-source; as such, its methodologies are transparent and reproducible, and the community can further expand the software.

Chipster is developed based on a client-server architecture. Data is imported at the client side, while all processing is performed on the server side using R programming. It requires that all data need to be transferred between client and server for each analysis step which can be very time-consuming if the datasets are large [22]. Chipster was not able to successfully perform a classification when we provided the dataset containing all probes. As a result, feature selection was performed artificially; that is, the datasets provided to Chipster contained only those probes selected by our CancerDiscover feature selection method; thus, datasets provided included the top 3, 6, 12, 100, or 500 probes. Raw data in the form of CEL files were normalized (RMA normalization) by Chipster. The accuracy using top 3 probes for the Tumor vs. Normal model was 97.63%, whereas, for the Adenocarcinoma vs. Squamous Cell Carcinoma model was 98.82%, ranking 3^rd^ for the accuracy assessment (See Supplementary Table 1 in Supplementary File 3). These accuracy assessments for the CancerDiscover are better than the results provided by Chipster.

Data used in this paper were also analyzed independently in Aliferis et al. [21], using two feature selection algorithms: Recursive Feature Elimination and Univariate Association Filtering. These algorithms identified 6 and 100 features, respectively, as significant for cancer vs. normal classification, and 12 and 500 features, respectively, for adenocarcinoma vs. squamous cell carcinoma classification. Aliferis et al. reported average accuracies across classification algorithms: 94.97% for cancer vs. normal model, and 96.83% for the squamous carcinoma vs. adenocarcinoma model. In comparison, CancerDiscover resulted in 99.21% accuracy for cancer vs. normal model, and 99.06% for the adenocarcinoma vs. squamous cell carcinoma model, while using only three features. In the context of these data, CancerDiscover was more accurate, while using less information than that of Aliferis et al.

These results demonstrate that the CancerDiscover method is complementary to some of the existing methods, such as GenePattern, Chipster, and Aliferis et al. methods, and that it is also suitable for accurate classification of other types of cancer types and subtypes. Although the classification accuracy of CancerDiscover was marginally higher than that of the compared methods, the strengths of CancerDiscover lie in its streamlined nature that enables users to begin with raw data and proceed to build machine learning models within a complete pipeline. Another strength of CancerDiscover is that it is flexible, allowing users to utilize various methodologies within the platform, and further extend the software as a whole due to its open-source nature.

In conclusion, we have developed a comprehensive, integrative, open-source, and freely available pipeline, CancerDiscover, which enables researchers to automate the processing and analysis of high-throughput data with the objective of both identifying cancer biomarkers and classifying cancer and normal tissue samples (including cancer sub-types). Herein, we showcased the pipeline's flexibility, utility, and ease-of-use in generating multiple prediction models simultaneously from raw high-throughput data. CancerDiscover allows users to customize each step of the pipeline, preprocessing raw data, selecting normalization methods, data partitions, feature selection algorithms, and classification algorithms for additional analysis. The CancerDiscover pipeline was able to identify an optimal number of top-ranked features and accurately classify the sample data into its classes. Benchmarking demonstrated the high performance of the pipeline across datasets of varying sizes. Researchers can now utilize CancerDiscover for diverse projects, including biomarker identification, and tissue classification without extensive technical knowledge while retaining significant flexibility. Another great advantage to the biomedical community is that unlike GenePattern and Chipster, CancerDiscover is open-source and freely available and written in a modular fashion which opens an array of opportunities for users to tweak the software themselves for their needs, adding more algorithms as it becomes available. Next, we will make our efforts to develop graphical user interface and web server for CancerDiscover with additional functionalities such as querying, searching and downloading datasets from the public repositories and data visualization of the outputs.

## MATERIALS AND METHODS

The presented integrative pipeline consists of existing open-source software tools and utilizes publicly available datasets and various performance metrics.

### Data collection

For the case study, lung cancer (tumor vs. normal and adenocarcinoma vs. squamous carcinoma) microarray gene expression data were collected from the Broad Institute Cancer Program Legacy Publication Resources database [23]. 237 tumor tissue samples and 17 histologically normal tissue samples, and 211 lung adenocarcinoma and squamous cell carcinoma samples were used. The plate used was Human Genome U95A oligonucleotide probe arrays, containing 12,625 probes. Benchmarking was performed using 500 samples of Acute Myeloid Leukemia (AML) and normal blood sample expression data downloaded from NCBI (GSE6891, GSE2677, GSE43346, GSE63270) [HG-U133_Plus_2] Human Genome U133 Plus 2.0 Array, containing 54,675 probes.

### Normalization and background correction

Normalization and preprocessing are essential steps for the analysis of high-throughput data. The Affy R module 1.54 [24] from Bioconductor package (https://bioconductor.org/packages/release/bioc/html/affy.html) was used to remove the technical variation from noisy data and background noise from signal intensities. This step is crucial for analyzing large amounts of data which have been compiled from different experimental settings where, individual data files are processed to remove sample bias from the data, which could otherwise introduce a bias in the model. Affy R package provides multiple methods for normalization and background correction, which can be utilized within CancerDiscover using programmatic flags. For the case study given above, quantile normalization [25] and robust multichip average (RMA) [20] were used for normalization and background correction, respectively.

### Machine learning algorithms and framework

Though the pipeline supports the diverse set of machine learning classifiers, we used Support Vector Machines (SVMs) and Random Forests to construct the models for the case study. These machine-learning methods were chosen because of their extensive and successful applications to datasets from genomic and proteomic domains [26, 27]. Some of the cancer classification tasks were binary (two classes), and the others were multiclass (more than two classes). Though SVMs are designed for binary classification, they can also be used for multiclass classification by a one-versus-rest approach [28]. The one-versus-rest approach for classification is known to be among the best-performing methods for multi-category classification for microarray gene expression data [29]. Models were also constructed using Random Forests (RF), which can solve multi-category problems natively through a direct application [30]. Waikato Environment for Knowledge Analysis (WEKA 3-6-11) [18] is a machine learning software environment that serves as a platform for clustering and classification of high-throughput data.

### Performance measure

Accuracy was defined as the overall ability of models to predict testing data correctly. Reported measures included the numbers of *true positives* (TP), *true negatives* (TN), *false positives* (FP), and *false negatives* (FN). A true-positive count is the number of samples in a dataset which were correctly categorized *into* classes. A false-positive count is the number of samples in a dataset which were sorted into the wrong category. A true negative count represents the number of samples which were *not* classified into a class to which they do *not* belong, and false negatives are samples which are *not* classified into the class to which they do belong. Accuracy, Sensitivity (or Recall), Specificity, Precision, F1-Score are derived from the measures mentioned above as follows: accuracy is the ratio of correctly predicted samples to the total number of samples. Sensitivity is the proportion of true positives that are predicted as positives. Specificity is the proportion of true negatives which are predicted as negatives, and Precision is the ratio of true positives to the total number of true negatives and true positives. Lastly, F1-score is defined as the harmonic mean of Precision and Recall and is calculated by first multiplying precision and recall values, then dividing the resulting value by the total of precision and recall, and finally, multiplying the result by two. The Accuracy, Sensitivity, Specificity, Precision, and F1-Score are given by:

$$Accuracy=\frac{TP+TN}{TP+TN+FP+FN}$$

$$Recall/Sensitivity=\frac{TP}{TP+FN}$$

$$Precision=\frac{TP}{TP+FP}$$

$$Specificity=\frac{TN}{TN+FP}$$

$$F1-Score=2*\frac{Precision*Recall}{Precision+Recall}$$

### Model selection and accuracy estimation

The pipeline offers the flexibility to choose any k-fold cross-validation for model selection and accuracy estimation. In the case study, we used stratified 10-fold cross-validation [27, 29]. This technique separates data into ten parts and uses nine parts for the model generation while predictions are generated and evaluated by using the one part. This step is subsequently repeated ten times, such that each part (internal test set) is tested against the other nine parts (internal train set). After the 10-fold cross-validation, the average performance of all of the folds is used as an unbiased estimate of the performance of model training.

## SUPPLEMENTARY MATERIALS TABLES







## Abbreviations

HTS: high throughput sequencing
HPC: high performance computing
SLURM: simple linux utility for resource management
WEKA: waikato environment for knowledge analysis
ML: machine learning
SVM: support vector machine
RF: random forests
RMA: robust multi average
TP: true positives
TN: true negatives
FP: false positives
FN: false negatives
AML: acute myeloid leukemia
ARFF: attribute relation file format.
