# Supplementary material for: CancerDiscover: an integrative pipeline for cancer biomarker and cancer class prediction from high-throughput sequencing data
Source: Oncotarget. 2017 Dec 20;9(2):2565–73. doi: 10.18632/oncotarget.23511 (PMC5788660; doi:10.18632/oncotarget.23511)
Supplement: Supplementary file 1 [file oncotarget-09-2565-s001.pdf]

CancerDiscover: an integrative pipeline for cancer biomarker and cancer class prediction from high-throughput sequencing data

SUPPLEMENTARY MATERIALS

Supplementary File 1: CancerDiscover User Manual for detailed installation/operation instructions

See Supplementary File 1

Supplementary File 2: List of the available feature selection methods

See Supplementary File 2

Supplementary File 3: Comparisons of CancerDiscover with other tools

Model	CancerDiscover	GenePattern	Chipster	Aliferis
Tumor vs. normal	99.21	98.43*	97.63*	94.97*
Adeno vs. squamous	99.06	99.06*	98.82*	96.83*

The accuracies of CancerDiscover, GenePattern, Chipster, and Aliferis.
*Due to the unavailability of analysis components, CancerDiscover components were used.
