# Supplementary material for: CancerDiscover: an integrative pipeline for cancer biomarker and cancer class prediction from high-throughput sequencing data
Source: Oncotarget. 2017 Dec 20;9(2):2565–73. doi: 10.18632/oncotarget.23511 (PMC5788660; doi:10.18632/oncotarget.23511)
Supplement: Supplementary file 2 [file oncotarget-09-2565-s002.docx]

**CancerDiscover Pipeline v. 1.0 User Manual**

**Purpose**

The purpose of this free, open-source pipeline tool is to allow users to efficiently and automatically process large high-throughput datasets by converting data (for example CEL files, etc.), normalizing, and selecting best performing features from multiple feature selection algorithms. The pipeline lets users apply different feature thresholds and various learning algorithms to generate multiple prediction models that distinguish different types and subtypes of cancer.

CancerDiscover v.1.0 is a collection of BASH, PERL, JAVA, AWK, and SLURM program files that can be run from the command line and a variety of text outputs will be generated. This documentation will provide a step-by-step procedure for generating cancer class prediction and biomarker (features) files. The pipeline is compatible with Linux OS and Mac OSX.

This README file will serve as a guide for using this software tool. We suggest reading through the entire document at least once, to get an idea of the options available, and how to customize the pipeline to fit your needs.

### **Table of Contents**

- [System Requirements](https://github.com/HelikarLab/CancerDiscover#system-requirements)
- [Downloading CancerDiscover and Dependencies](https://github.com/HelikarLab/CancerDiscover#downloading-cancerdiscover-and-dependencies)
- [Directory Structure of the Pipeline](https://github.com/HelikarLab/CancerDiscover#directory-structure-of-the-pipeline)
- [Execution of Pipeline](https://github.com/HelikarLab/CancerDiscover#execution-of-pipeline)
- [Contribution](https://github.com/HelikarLab/CancerDiscover#contribution)
- [License](https://github.com/HelikarLab/CancerDiscover#license)

**System Requirements**

You will need current or very recent generations of your operating system. Linux OS, Mac OS X.

**Downloading CancerDiscover and Dependencies**

curl -sL bit.do/installation_linux | sh

curl -sL bit.do/installation_mac | sh

**Directory Structure of the Pipeline**

After downloading CancerDiscover, notice inside the CancerDiscover directory there are several empty directories and one which contains all of the scripts necessary to process data.

***DataFiles*** directory contains raw data files and sampleList.txt file

***Outputs*** *repository* contains “resultsSummary.txt” file which will have the summary of the model accuracies as well as information regarding the context which gave the highest accuracy.

***Scripts*** directory contains all of the source code

***Models*** repository contains all of the classification models

***Temp*** directory contains intermediate files that are generated as part of the execution of the pipeline

***Feature Selection*** directory contains the feature selection algorithm output files and two nested directories for arff file generation, namely Chunks and ArffPreprocessing

***Chunks*** contains different threshold feature sets

***ArffPreprocessing*** directory contains the feature vectors in arff format. Feature vectors made here are split into training and testing datasets in their respective directories.

***Train*** is the repository of the training data for the modeling

***Test*** is the repository of the testing data for model testing

***SampleData*** is a directory which contains 10 sample files and their associated sampleList.txt file

***Logs*** is a directory which contains the elapsed time in seconds for each leg of the pipeline from initialization through model testing.

***CompletedExperiments*** When the pipeline has finished running, the above directories which contain experimental data will be moved into this directory. This a directory will act as a repository of old experiment files organized by a time-stamp which reads Year, month, day, hours, minutes, seconds; in that order

**Execution of Pipeline**

The first step is to place your raw CEL files into the DataFiles Directory.

In the DataFiles directory you will need to create a two-column csv (comma separated file) called "sampleList.txt" in the first column write the name of each raw data file, and in the second column write the class identifier to be associated with that sample.

If you want to use the sample data for classification:

**cp SampleData/* ../DataFiles**

This command will copy all of the data and sampleList.txt files in the SampleData directory to the DataFiles directory.

**Overall, the users will require to run only 4 scripts**

**bash initialization.bash** # Initialization

**bash masterScript_1.bash** # Normalization

**bash masterScript_2.bash** # Feature Selection

**bash masterScript_3.bash** # Model Training and Testing

1. **Initialization**

Once you have finished making the sampleList.txt file in the DataFiles directory, please go inside the “Scripts” directory to execute the next steps of the pipeline. There are two versions of the pipeline, BASH, and SLURM (Simple Linux Utility for Resource Management). SLURM is a computational architecture used to organize user requests into a queue to utilize super-computer resources. SLURM requires no kernel modifications for its operation and is relatively self-contained. Depending on your access to a SLURM scheduler, you will use one or another set of scripts. If you do have access to a SLURM scheduler you will execute the scripts ending in “.slurm”. Otherwise, you will use the scripts ending in “.bash”. Due to the complexity of data manipulation, and/or the sheer size of your data, it is recommended to use a high-performance computer.

Now, in the scripts directory, edit the file called “Configuration.txt”, to make any changes desired for processing your data including the normalization method, the size of data partitions, and which feature selection and classification algorithms are to be executed.

The default settings for normalization are:

Normalization method="quantiles",

Background correction method="rma",

Pm value correction method="pmonly",

Summary method="medianpolish"

The default setting for data partitioning is 50:50. The default setting for feature selection algorithms will perform all possible feature selection algorithm options. You can find the list of feature selection methods and their associated file names in the “Scripts” directory in the file named “featureSelectionAlgorithms.lookup”. The default setting for classification algorithms will generate models using the following algorithm options; the Decision Tree, IBK, Naive Bayes, Random Forest, and Support Vector Machine. If you wish to use other classification algorithms than the ones provided, refer to the WEKA resources at weka.wikispaces.com/Primer. In the configuration file, you will also need to write in the absolute path. This path should end in CancerDiscover; for example, a directory path might look like: work/userGroup/userMember/data/CancerDiscover

**cd ../Scripts**

**bash initialization.bash**

1. **Normalization**

**bash masterScript_1.bash**

**For SLURM users:**

**sbatch masterScript_1.slurm**

The purpose of the above script is to perform normalization on raw data and generate the Expression set matrix.

1. **Feature Selection**

After normalization is complete, you will have a single file called “ExpressionSet.txt” in your DataFiles directory. The next step is to build a master feature vector file using the ExpressionSet.txt file. The next command you use will build this master feature vector file for you using the ExpressionSet.txt file, as well as perform data partitioning, or divide the master feature vector file into two parts; training and testing. The program will then perform feature selection using only the training portion of the master feature vector. Additionally, you can find the list of feature selection methods and their associated file names in the “Scripts” directory in the file named “featureSelectionAlgorithms.lookup”.

The default setting for data partitioning is 50/50, meaning the master feature vector file will be split evenly into training and testing data sets while retaining approximately even distributions of your sample classes between the two daughter files. To achieve a larger split, such as 80/20 for training/testing, in the configuration file, Configuration.txt, replace the 2 with a 5. This will tell the program to perform five folds, where the training file will retain four and the testing file will retain a single fold or 20% of the master feature vector data.

The default setting for feature selection will perform all possible forms of feature selection available unless otherwise specified in the “configuration.txt” file. If you wish to change these feature selection options, in the “Scripts” directory you will need to edit the file named “configuration.txt”. Simply write “TRUE” next to all of the feature selection methods you wish to perform and “FALSE” if you do not want that method performed. Additionally, you can find the list of feature selection methods and their associated file names in the “Scripts” directory in the file named “featureSelectionAlgorithms.lookup”.

The following commands perform the feature selection from normalized expression matrix:

**bash masterScript_2.bash**

**For SLURM users:**

**sbatch masterScript_2.slurm**

1. **Model Training and Testing**

Once feature selection has been completed, new feature vectors are made based on the ranked lists of features. The new feature vectors will be generated based on your threshold selections, and immediately used to build and test classification models using a classification algorithm of your choosing. Lastly, the directories will be reset, and your old directories and files will be placed in the “CompletedExperiments” followed by a timestamp.

The last lines of the masterScript_3 scripts will move the content of the “DataFiles” to “CompletedExperiments” so the new experiment will run in DataFiles directory. You can find all raw data, feature selection outputs, training and testing feature vectors, models, and model results in the “CompletedExperiments” directory followed by a time-stamp. To run experiments with new data, begin with step 1.

**bash masterScript_3.bash**

**For SLURM users:**

**sbatch masterScript_3.slurm**

**Contribution**

Akram Mohammed [amohammed3@unl.edu](mailto:amohammed3@unl.edu)

Greyson Biegert [greyson@huskers.unl.edu](mailto:greyson@huskers.unl.edu)

Jiri Adamec [jadamec2@unl.edu](mailto:jadamec2@unl.edu)

Tomas Helikar [thelikar2@unl.edu](mailto:thelikar2@unl.edu)

**License**

This software has been released under the [GNU General Public License v3](https://github.com/HelikarLab/CancerDiscover/blob/master/LICENSE.md).
