# Supplementary material for: CancerDiscover: an integrative pipeline for cancer biomarker and cancer class prediction from high-throughput sequencing data
Source: Oncotarget. 2017 Dec 20;9(2):2565–73. doi: 10.18632/oncotarget.23511 (PMC5788660; doi:10.18632/oncotarget.23511)
Supplement: Supplementary file 3 [file oncotarget-09-2565-s003.docx]

**#This is a lookup file to standardize the names of the feature selection algorithm**

**%Feature Selection Algorithm + Search Method Combinations%**

1 Cfs Subset Evaluator + Best first

2 Cfs Subset Evaluator + Genetic Search

3 Cfs Subset Evaluator + Greedy Stepwise

4 Cfs Subset Evaluator + Linear Forward Selection

5 Relief F Attribute Evaluator + Ranker

6 Wrapper Subset Evaluator + Subset Size Forward Selection

7 Chi Squared Attribute Evaluator + RankeR

8 Wrapper Subset Evaluator + Rank Search

9 Classifier Subset Evaluator + Genetic Search

10 SymmetricalUncertAttributeEval + Ranker

11 Classifier Subset Evaluator + Linear Forward Selection

12 Classifier Subset Evaluator + Rank Search

13 Classifier Subset Evaluator + Subset Size Forward Selection

14 Consistency Subset Evaluator + Best first

15 Consistency Subset Evaluator + Genetic Search

16 Wrapper Subset Evaluator + Genetic Search

17 Consistency Subset Evaluator + Linear Forward Selection

18 Consistency Subset Evaluator + Rank Search

19 Consistency Subset Evaluator + Subset Size Forward Selection

20 Filtered Attribute Evaluator + Ranker

21 One R Attribute Evlauator + Ranker

22 Latent Semantic Analysis + Ranker

23 Gain Ratio Attribute Evaluator + Ranker
